# Neuropilins, as Relevant Oncology Target: Their Role in the Tumoral Microenvironment

**DOI:** 10.3389/fcell.2020.00662

**Published:** 2020-07-17

**Authors:** Aurore Dumond, Gilles Pagès

**Affiliations:** ^1^Medical Biology Department, Centre Scientifique de Monaco, Monaco, Monaco; ^2^Inserm U1081, CNRS UMR 7284, Centre Antoine Lacassagne, Institut de Recherche sur le Cancer et le Vieillissement de Nice, Université Côte d’Azur, Nice, France

**Keywords:** neuropilins, tumor microenvironment, oncology, immunology, cancers

## Abstract

Angiogenesis is one of the key mechanisms involved in tumor growth and metastatic dissemination. The vascular endothelial growth factor (VEGF) and its receptors (VEGFR) represent one of the major signaling pathways which mediates angiogenesis. The VEGF/VEGFR axis was intensively targeted by monoclonal antibodies or by tyrosine kinase inhibitors to destroy the tumor vascular network. By inhibiting oxygen and nutrient supply, this strategy was supposed to cure cancers. However, despite a lengthening of the progression free survival in several types of tumors including colon, lung, breast, kidney, and ovarian cancers, modest improvements in overall survival were reported. Anti-angiogenic therapies targeting VEGF/VEGFR are still used in colon and ovarian cancer and remain reference treatments for renal cell carcinoma. Although the concept of inhibiting angiogenesis remains relevant, new targets need to be discovered to improve the therapeutic index of anti-VEGF/VEGFR. Neuropilin 1 and 2 (NRP1/2), initially described as neuronal receptors, stimulate angiogenesis, lymphangiogenesis and immune tolerance. Moreover, overexpression of NRPs in several tumors is synonymous of patients’ shorter survival. This article aims to overview the different roles of NRPs in cells constituting the tumor microenvironment to highlight the therapeutic relevance of their targeting.

## Generalities on the Neuropilins

### Genomic Organization and Protein Structure

The Neuropilins are type-1 membrane glycoproteins of 130–140 kDa. Two proteins of the same family, Neuropilin 1 and 2 (NRP1 and NRP2), coded by two different genes on independent chromosomes (10p12 for NRP1 and 2q34 for NRP2), share 44% of sequence homology. They are composed of a N-terminal extracellular domain, a transmembrane domain and a cytoplasmic domain of 43–44 amino acids. The extracellular domain comprises five subdomains: a1, a2, b1, b2, and c. The cytoplasmic part does not contain a signaling domain but has a PDZ domain and a triplet of amino acids “serine, glutamic acid, alanine (SEA).” The PDZ domain enables the formation and the stimulation of signaling complexes. The membrane and cytoplasmic parts are implicated in the receptors’ dimerization. Soluble forms of NRP1 and NRP2 (sNRP1, sNRP2) without transmembrane and without cytoplasmic domain and an isoform of NRP2 without the SEA amino acid triplet are formed after alternative splicing.

### The Phenotype of Knock-Out Mice

NRP1 gene invalidation (KO) induces defects in vascular, nervous, and cardiac network and leads to an embryonic lethality between 10 and 12.5 days (Kawasaki et al., 1999). The overexpression of NRP1 is lethal for embryos of about 12.5 days with cardiac defects (Kitsukawa et al., 1995).

NRP2 KO is not lethal but a diminution of lymphatic vessels and some abnormalities during the neural development are observed (Yuan et al., 2002).

Mice with a double NRP1 and NRP2 KO present more severe vascular abnormalities and embryos die at 8.5 days (Takashima et al., 2002) with the presence of important avascular zones and of some gaps between the blood vessels.

### NRP Ligands

The NRPs bind to specific ligands and form heterodimers with five families of receptors. The dimerized ligands bind to the NRP homo- or heterodimers and to partner receptors dimers to form a complex which induces a specific intracellular signal. The sNRP are competitive forms for the binding of vascular endothelial growth factor (VEGF) to the membrane NRP1.

#### SEMA3/Plexin

The NRPs were first described as neuronal receptors binding the semaphorins (SEMA, seven classes described) which constitute a family of proteins that guide axons growth and are involved in cell apoptosis, migration and tumor suppression. SEMA3C is involved in endothelial cell apoptosis, it inhibits pathological angiogenesis and it promotes invasion and metastasis in cancers. SEMA3A is an angiogenesis inhibitor, that is less expressed during tumor development. Indeed, it controls pericytes recruitment to vessels (Niland and Eble, 2019). Neuropilins form a complex with SEMA receptors, the plexins. The binding of the SEMA on NRP is established through the a1, a2, b1, and b2 domains (Roy et al., 2017). The ternary complex between NRPs, SEMAs and the plexins enhances signal transduction during development, axon guidance and immunity. NRP1 binds preferentially to SEMA3A and NRP2 to SEMA3C or 3F (Roy et al., 2017). SEMA3E/PlexinD1 pathway is involved in the initial development of axon tracts in the forebrain and in the establishment of functional neuronal networks. Some axons expressed plexinD1 but not NRP1, in this case SEMA3E acts as a repellant. When neurons express plexinD1 and NRP1, SEMA3E is an attractant (Chauvet et al., 2007). The extracellular part of NRP1 is sufficient in inducing the attractive axonal guidance. PlexinD1 is necessary for SEMA3E’s effects on axonal guidance. However, NRP1 is necessary to control the gating response of SEMA3E to induce a repulsive or attractive axon growth (Chauvet et al., 2007). According to the major role played by the NRP1/SEMA3E signaling in neurodevelopment, any defect may be related to neural disorder as it was suggested in a mouse model of schizophrenia (Daoust et al., 2014).

#### VEGF/VEGFR

The VEGF gene is composed of eight exons. Exons 1–5 are implicated in the binding to vascular endothelial growth factor receptors (VEGFR) and exons 7 and 8 in the binding to NRP1 and NRP2 (Guyot and Pages, 2015). The differential splicings of exon 6, 7 and 8 induce two distinct families of isoforms. Isoforms with the exon 8a are pro-angiogenic and isoforms with exon 8b are anti-angiogenic (Harper and Bates, 2008). Four predominant forms of VEGF exist: VEGF121, VEGF189, VEGF206 and the more abundant and active in many cancers, the VEGF165. The VEGF165 binds preferentially to NRP1 (Kd = 0.2 nM) as compared to NRP2 (Kd = 5 nM).

In healthy people, VEGFs are involved in wound healing and vascular homeostasis. However, VEGFs promote tumor angiogenesis and lymphangiogenesis and high levels of VEGFs expression are synonymous of poor prognosis in cancers. NRP1 binds the VEGF165 and the receptors VEGFR1 and 2. VEGF binding stimulates this pathway leading to increased angiogenesis. NRP2 binds the VEGF165 and VEGFC, the main lymphangiogenic factor, and forms a complex with the receptors VEGFR2 or VEGFR3 to stimulate angiogenesis and lymphangiogenesis. The binding occurs through the NRPs’ b1 and b2 domains. VEGFR activation by the VEGF does not require the NRP. However, in some tumors, VEGFRs are absent and NRP1 induce cell migration and angiogenesis in a VEGFR-independent manner. VEGF binding to NRP1, independently of VEGFR, activates RhoA and Ras, two effectors of different signaling pathways (Niland and Eble, 2019).

Thus, the stimulation of NRP by the VEGF is highly relevant in a therapeutic context.

#### PlGF/VEGFR

Placenta growth factor (PlGF) belongs to the VEGFs family and binds to VEGFR1 but not to VEGFR2. It was initially described as a placenta produced homodimeric protein. Three isoforms are initiated from alternative splicing: PlGF1, PlGF2, and PlGF3. PlGF2 is the only form containing exon 6, which codes for an heparin binding domain (Migdal et al., 1998). PlGF2 binds to NRP1 through amino acids encoded by exon 6 and exon 7 and PlGF1 through amino acids encoded by exon 7 (Migdal et al., 1998). In breast cancer, PlGF1 and NRP1 overexpression is correlated to a poor prognosis and PlGF2 is overexpressed in cancer tissues as compared to normal tissue (Escudero-Esparza et al., 2010). The PlGF/NRP pathway is implicated in tumor growth, angiogenesis, migration, and metastasis for melanoma cancers even in the absence of VEGFRs (Pagani et al., 2016). PlGF is also a relevant target in retinal diseases resistant to anti-VEGF therapies (Van Bergen et al., 2019). In the Sonic Hedgehog subgroup of medulloblastoma, PlGF binds to NRP1 leading to mitogen activated protein kinase (MAPK) signaling activation, tumor growth and dissemination (Snuderl et al., 2013). Moreover, the PlGF/NRP signaling pathway plays a key role in resistance to anti-angiogenic therapies (Pagani et al., 2016).

#### HGF/cMET

The signaling pathway induced by the hepatocyte growth factor (HGF) and its receptor (cMET) regulates endothelial cell survival, proliferation and migration. HGF/cMET complex plays an important role in tumor progression. NRP1, by binding to cMET, induces tumor invasion. As HGF/cMET inhibits apoptosis and promotes immune tolerance by interacting with the programmed death ligand 1 (PD-L1) (Balan et al., 2015), the stimulation of this signaling pathway by NRP1 promotes tumor growth by inhibiting the antitumor immunity.

#### TGFβ1/TGFβRs

TGFβ1/TβRs stimulates the SMAD2/3 signaling pathway, which is involved in physiological development, host immunity, inflammation and in tumor progression, and invasion. TGFβ also promotes cancer progression and metastasis (Chaudhary et al., 2014). TGFβ binds to NRP1 via its b1 domain and forms a complex with TGFβ receptors I–III. Activation of this signaling pathway stimulates angiogenesis in a VEGFR2-independent manner. NRP1/TGFβ/TGFβR also promotes T regulatory lymphocytes activity and immune tolerance.

#### PDGF/PDGFR

The increased expression of PDGF and its receptors on tumor vasculature promotes pathological angiogenesis (Chaudhary et al., 2014). This signaling pathway also induces cell proliferation, differentiation, and epithelial to mesenchymal transition (Niland and Eble, 2019). Four PDGF variant exist: PDGFA, B, C, and D. These ligands bind to the tyrosine-kinase receptors PDGRFα or β. Depending on the ligand, the receptors will homo- or hetero-dimerize giving three possible combinations: αα, αβ, or ββ. PDGF-stimulated PDGFRs activate MAPK and PI3K signaling pathways. NRP1 forms a complex with PDGF and PDGFR amplifying their respective downstream signaling pathways.

#### FGF/FGFR2

FGF/FGFR2 complex induces cell migration and proliferation. This axis is key for endothelial cell proliferation and subsequent angiogenesis. By forming a complex with the FGFR2, the NRPs play a key role in amplifying its signaling pathways and consequently these biological phenomena.

#### Galectins

Galectins, part of the family of β-galactoside-binding proteins, are involved in cell-cell and cell-matrix interactions. Galectin-1 (Gal-1) induces tumor-associated HuVEC proliferation and migration, by enhancing VEGFA effects, and HuVEC adhesion. Gal-1 exerts these effects through VEGFR2 phosphorylation enhanced by Gal-1/NRP1 binding (Hsieh et al., 2008). The activation of NRP1/VEGFR1-dependent AKT signal by Gal-1 decreases endothelial-cadherin cell-cell junctions and increases the vascular permeability (Wu et al., 2014).

#### EGF/EGFR

Epidermal growth factor receptor (EGFR) is a monomeric transmembrane protein. EGFR mutations were described in several forms of cancers, such as breast or lung cancers and it is overexpressed in numerous tumors. EGFR activation stimulates AKT signaling. NRP1 extracellular domain is necessary for EGFR-endocytosis and AKT-dependent cancer cell viability and tumor growth. Hence, reduced expression of NRP1 limits EGFR endocytosis (Rizzolio et al., 2012). Furthermore, NRP2 is required, through WDFY1 (WD-repeat and FYVE-domain-containing protein 1), to activate EGFR endocytosis in cancer cells and to maintain EGFR activities (Dutta et al., 2016).

#### Hedgehog Signaling Pathway

This pathway is involved in embryogenesis and in adult’s tissue healing. Its activation induces cell proliferation and differentiation. Its overexpression or downregulation induces cancer development and the epithelial-mesenchymal transition (EMT). NRPs are major regulators of the Hedgehog signaling pathway. A feedback loop exists between NRP1 and Hedgehog; Hedgehog signaling induces NRP1 expression, which promotes activation of Hedgehog targeted gene (Niland and Eble, 2019). A down-regulation of NRP1 by shRNA in ccRCC cell lines reduces sonic hedgehog (SHH) and its activator Gli1 expressions. SHH signaling pathway inhibition promotes tumor cell differentiation (Cao et al., 2008).

#### Integrins

NRPs also interacts with integrins. The intercellular interaction between integrins α5β1 and α9β1 expressed on endothelial cells and NRP2 expressed on tumor and endothelial cells increases tumor spreading and metastasis through and integrin-dependent mechanism (Cao et al., 2013; Alghamdi et al., 2020).

## The Role of Neuropilins in the Immune System (Figure 1)

### Dendritic Cells

They are recruited to the tumor site. After their contact with the antigen, they are maturated, which enables them to migrate to the lymphoid organs to activate naïve T cells and to induce the primary immune response. Two types of dendritic cells (DCs) exists: (i) myeloid DCs (mDCs) that present the antigen to T cells; (ii) plasmacytoid DCs (pDCs), generally involved in immune suppression. Activated pDCs have an antigen presenting capacity, they also activate T cells but to a lesser extent as compared to mDCs.

**FIGURE 1:**
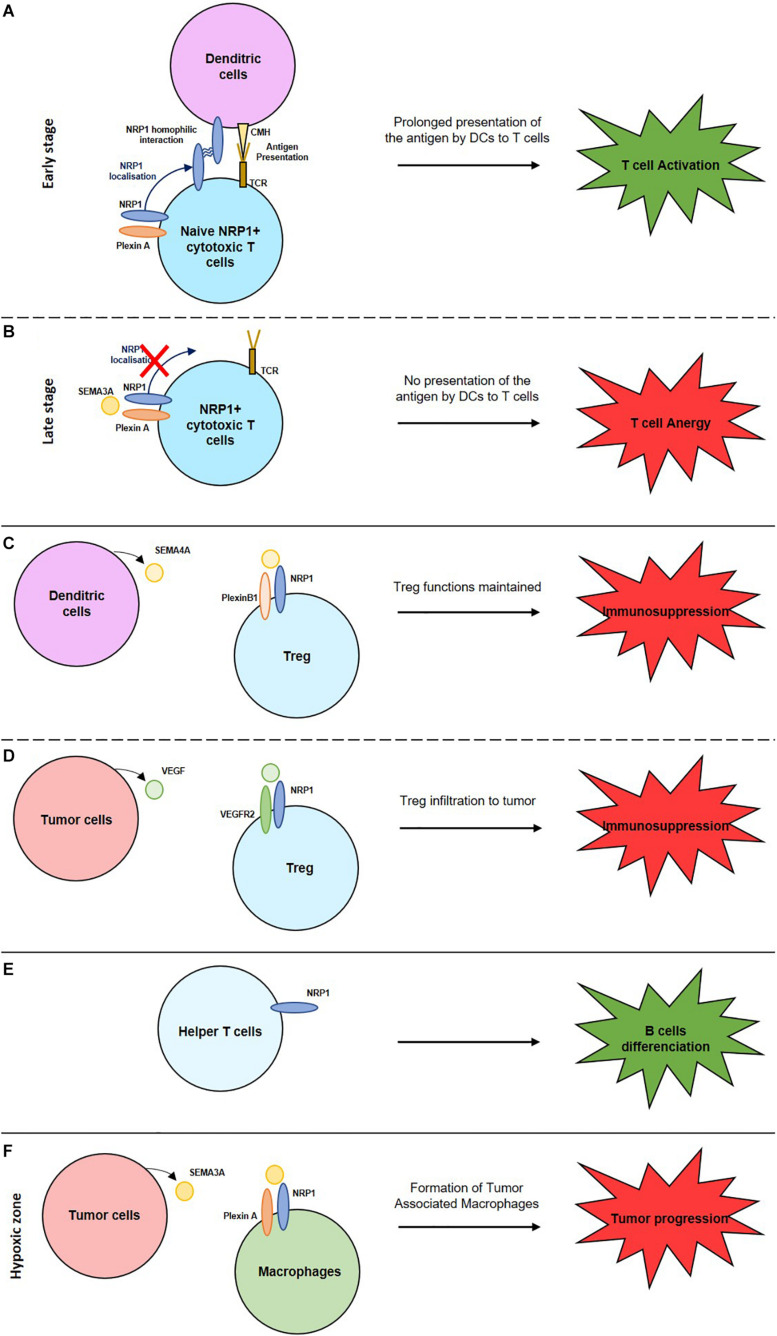
Role of NRPs in the activation or suppression of the immune system. **(A)** NRP1 homophilic interaction enhances the interaction between naive cytotoxic T cells and dendritic cells inducing a prolonged antigen presentation and so T cell activation. **(B)** Expression of SEMA3A by mature cytotoxic T cells inhibits NRP1 localisation and induces T cell anergy. **(C)** Interaction between NRP1, expressed by Treg cells, with SEMA4A, expressed by dendritic cells maintain Treg functions. **(D)** Interaction between NRP1, expressed by Treg cells, with VEGF, expressed by tumor cells, enable Treg cells infiltration into the tumor and induce an immunosuppression. **(E)** NRP1+ helper T cells induce B cells differentiation to activate their immune response. **(F)** Interaction between NRP1, expressed by macrophages, with SEMA3A, expressed by tumor cells, induce the formation of tumor associated macrophages (TAM) and so a tumor progression.

NRP1 is expressed on mature DC and on naïve T cells. This enables NRP1/NRP1 homophilic interaction and the formation of an immunological synapse between these two cell types. Thus, NRP1 mediates the primary immune response activation by promoting antigen presentation by DCs through this synapse (Sarris et al., 2008; Akkaya et al., 2019). NRP1 regulate cytoskeleton rearrangements allowing their transmigration to the lymphatics and lymphoid tissues to activate T cells. However, at a late stage of T cell activation, SEMA3A is secreted. By its interaction with NRP1 expressed on T cells, it disrupts the formation of the immunological synapse with the DC resulting in reduced T cell activation and immune tolerance (Lepelletier et al., 2006).

NRP2 expression increases during the differentiation from monocytes to dendritic cells (Schellenburg et al., 2017). Its sialylation protects DC during their migration to lymph nodes. In the lymph nodes, the polysialic acid is eliminated of NRP2 and DC activate T cells (Curreli et al., 2007; Rey-Gallardo et al., 2011).

### Macrophages

They play a key role in immune surveillance, in cellular debris elimination and in antigen presentation. M1 macrophages are pro-inflammatory and M2 are pro-angiogenic, immunosuppressive, thus pro-tumoral particularly in hypoxic zones. Hypoxia induces SEMA3A expression on tumoral cells. It interacts with NRP1, and their receptors plexin A1 and A4, expressed on macrophages. Tumor-associated macrophages (TAM) reside in the hypoxic zones where they exert their pro-tumoral role. If NRP1 expression decreases, TAM remain in the normoxic peripheric zones of the tumor resulting in the suppression of their pro-tumoral role (Casazza et al., 2013; Chen et al., 2019). In the microglia, NRP1 plays an immune suppressive role by inducing a M2 phenotype. A NRP1/NRP1 homophilic interaction with the helper T cells induces immune suppression. NRP1 expression on glioma-associated macrophages (GAM) induces a pro-tumoral response. NRP1 inhibition reduces tumor growth and a macrophages polarization to an anti-tumoral role (Cherry et al., 2014; Caponegro et al., 2018).

NRP2 expression increases during the differentiation of monocytes to macrophages (Schellenburg et al., 2017) next to inflammatory zones to induce phagocytosis. NRP2 sialylation reduces phagocytosis capacity of the macrophages (Stamatos et al., 2014; Roy et al., 2018), thus NRP2+ M2 macrophages promote tumor progression (Niland and Eble, 2019).

### T Cells

They are responsible of the adaptative immune response required for the control and the elimination of pathogenic agents and of tumor cells. Any dysfunctions in their development or activation induce auto-immune diseases and cancers. NRP1 is upregulated on active T cells (Chaudhary et al., 2014). Four types of T cells exist.

#### Cytotoxic T Cells (T CD8+)

They destroy the infected cells presenting the specific antigen through the class I major histocompatibility complex (MHC). The NRP1 expression is increased on CD8+ effective and memory T cells and promotes the antigen recognition (Roy et al., 2017). However, the exact NRP1 role in this context is unknown. NRP1 expression also correlates with PD1 expression on CD8+ T cells. Thus, NRP1 might represent a relevant biomarker to determine the efficacy of anti-PD1 immunotherapies. Indeed, patients with non-small cell lung cancer invaded with PD1-positive CD8+ T cells are highly responsive to anti-PD1 immunotherapies and present a longer survival (Leclerc et al., 2019).

#### Helper T Cells (T CD4+)

They are not cytotoxic but produce interleukin 2 and interferon gamma. These cytokines stimulate T and B cell proliferation. NRP1 is expressed on CD4+ T cells and induces B cells differentiation (Roy et al., 2017). Induction of NRP1 on regulatory T cells (Bruder et al., 2004) and on CD4+ T cells (Campos-Mora et al., 2019) induces immunosuppressive functions *in vivo*.

#### NKT Cells

They constitute a link between innate and adaptative immunity. Once activated, they lyse the targets and produce anti- and pro-inflammatory cytokines. NRP1 role on these NKT cells is unknown (Roy et al., 2017).

#### Regulatory T Cells (Treg)

Tregs play a role in immune homeostasis, allergic responses, auto-immune diseases, tumor immunity, and graft rejection. Their accumulation in tumors induces cancer progression and immune suppression (Sakaguchi et al., 1995). NRP1 is overexpressed by activated Tregs and promote their immunosuppressive role. NRP1 expression maintains the Tregs functions through the binding to SEMA4A, expressed by dendritic cells. NRP1/SEMA4A binding stabilizes the Treg by recruiting PTEN (Phosphatase and tensin homolog) and by inhibiting AKT phosphorylation. NRP1 expression on Treg induces their migration to the tumors where they play an immune-suppressive role (Hansen et al., 2012) by secreting IL-10 and IL-35, an anti-inflammatory cytokine. NRP1 expressed by Tregs are also attracted to tumors expressing VEGF where NRP1 acts as a VEGF co-receptor. The stimulation by VEGF, enhances T regs infiltration to tumors and an immunosuppressive response (Hansen et al., 2012).

CD4+/CD8+ T cells over-express NRP2 but NRP2 expression is lower on T cells expressing only CD8 or only CD4. The interaction between NRP2, SEMA3F and plexinA1 inhibits immature T cell migration.

Thus, the NRP have different roles in the immune system either in cell migration, cell-cell interaction or in the regulation of the immune response.

## Roles in Cancer

Neuropilins expression level correlates with tumor growth, invasiveness, angiogenesis, and poor prognosis. NRPs over-expression is often observed in carcinoma, melanoma, glioblastoma, leukemia, and lymphoma in which NRPs exert diverse functions.

### Functions of Neuropilins in Cancer

To grow over a few millimeters tumors turn into a pro-angiogenic environment that induces the formation of new blood vessels from the existing vascular network. This new vascular network surrounding the tumor, supplies oxygen and nutrients needed for tumors growth. Tumor cells, cells from the microenvironment and NRPs expressed on both cell types influence tumor angiogenesis (Niland and Eble, 2019). The roles of NRP1 in the growth and invasiveness of prostate, colorectal, kidney, lung, breast, … human cancers have been confirmed with animal studies showing that exacerbated angiogenesis and a poor prognosis is correlated with NRP1 expression (Ellis, 2006). Only in pancreatic cancers, a high expression of NRP1 correlates with reduced vascularized areas, decreased tumor growth, and improved survival (Morin et al., 2018). Expression of NRP2 is mostly correlated to tumor progression. In most cancers, the co-expression of NRP1 and NRP2 stimulates tumor growth and invasiveness (Rizzolio and Tamagnone, 2011). SEMA3C, which binds to NRP1 and NRP2 with equivalent affinity, inhibits tumor lymphangiogenesis by targeting immature vessels sprouting. However, its cleaved form, p65-SEMA3C, stimulates tumor lymphangiogenesis and metastatic dissemination of cancer cells expressing NRP2 (Mumblat et al., 2015).

Neuropilin 1 expression on tumor cells enhances cell viability, proliferation, migration, metastasis and favors cancer cell stemness. Since NRP1 promotes EMT through different pathways (TGF-β, Hedgehog, HGF…), which explains NRP1’s pro-tumoral role.

Neuropilin 1 is expressed on breast cancer cells, and its interaction with VEGF165 inhibits apoptosis. Such inhibition is counteracted by SEMA3B (Ellis, 2006). SEMA3F competes with VEGF in binding to the NRPs and blocks breast cancer cell migration. However, SEMA3F decreases membrane E-cadherin, which promotes cell metastasis (Ellis, 2006). SEMA3A expressed on endothelial cells, antagonizes VEGF effects and correlates with a good prognosis (Niland and Eble, 2020). It is generally lost during tumor progression (Niland and Eble, 2019). In a VEGFA+/SEMA3A+ environment, NRP1 binds preferentially SEMA3A (Palodetto et al., 2017). Cells with a higher VEGF expression as compared to SEMA3A expression have promigratory characteristics.

In colon cancer, NRP1 expression correlates with increased vessel number and poor prognosis, while NRP2 over-expression stimulates tumor progression and the down-regulation of NRP2 expression inhibits tumorigenesis and increases apoptosis (Gray et al., 2008). In prostate cancer, elevated NRP1 levels stimulated by VEGF inhibit tumor cell apoptosis and angiogenesis and are synonymous of shorter survival. In ccRCC, NRP1 down-regulation reduces migration, invasion, and tumorigenesis (Cao et al., 2008), and NRP2 down-regulation decreases cell extravasation in the lymphatic network and the metastatic spread (Cao et al., 2013). NRP1 expression down-regulation in experimental model of lung cancer reduces cell migration, invasion, and metastasis (Hong et al., 2007).

### Role in Cancer Stem Cells

A tumor is composed of cells differing in their morphology, their capacity to proliferate and to form metastasis and in their resistance to therapeutic agents. Among these different cells, only cancer stem cells (CSCs) are able to initiate a new primary tumor or metastasis. CSCs are cells that self-renew and that induce the heterogeneous aspect of the tumors. CSCs are resistant to chemo-and radiotherapy. As NRPs are less expressed in epithelial tissues compared to carcinomas, NRPs might play a role in stemness.

The role of the VEGFs/NRPs pathways have been studied in the triple negative breast cancer cell line MDA-MB-231 and the hormone sensitive MCF-7 cell line. While MDA-MB-231 have stemness characteristics MCF-7 cells have low stemness properties. In these cells, the level of stemness was correlated to the expression of VEGF and NRP1 (Zhang et al., 2017). Down-regulation of VEGF and NRP1 in MDA-MB-231 cells and overexpression of VEGF and NRP1 in MCF-7 cells confirmed that the VEGF/NRP1 signaling pathway is instrumental in driving stemness properties of breast cancer cells (Zhang et al., 2017). The VEGFC/NRP2 pathway is also involved in breast cancer stemness (Wang et al., 2014). The VEGF/NRP2 pathways stimulates stemness through activation of the YAP/TAZ signaling (Elaimy and Mercurio, 2018). This pathway also mediates homologous recombination by stimulating Rad51 expression leading to resistance to platinum chemotherapy in triple negative breast cancers (Elaimy et al., 2019). The NRP2/α6β1 integrin interaction activates the focal adhesion kinase (FAK) involved in tumorigenesis and associated to aggressive tumors (Goel et al., 2013). Furthermore, the VEGF/NRP1 pathway induces CSCs in breast cancers by activating the Wnt/β-catenin pathway (Zhang et al., 2017), which is involved in the induction of CSCs. The implication of VEGF/NRP1 pathway was also highlighted in glioma stem cells (Hamerlik et al., 2012) and in medulloblastoma stem cells (Gong et al., 2018).

### Role in Cancer-Associated Fibroblasts

Fibroblasts are part of the tumor microenvironment and become myofibroblasts (normal activated fibroblasts) under tumoral conditions. By interacting with fibronectin, myofibroblasts promote fibronectin fibril assembly, and tumor growth through α5β1 integrin (Yaqoob et al., 2012). Fibronectin fibril assembly is regulated determinant of matrix stiffness involved in tumor progression. NRP1 induces integrin function by binding to fibronectin and by activating the intracellular kinase c-Abl (Yaqoob et al., 2012). Indeed, NRP1 intracellular domain stimulates c-Abl that activates small GTPases (Rac or Rho). These GTPases promote α5β1 integrin function and so increase fibronectin binding and assembly (Yaqoob et al., 2012). The NRP1 extracellular domain is O-linked glycosylated via the serine 612 residue, which increases NRP1 binding to fibronectin resulting in enhanced fibronectin and α5β1 integrin interaction (Yaqoob et al., 2012). Thus, NRP1 intra- and extracellular domains, through the activation of c-Abl and α5β1 integrin, increase fibronectin fibril assembly contributing to matrix stiffness and tumor progression and invasiveness. Furthermore, cancer-associated fibroblasts (CAFs) are one of the most expressed cells in the tumor microenvironment, and the principal source of TGFβ1. NRP1/TGFβ1 interaction stimulates endothelial-mesenchymal transition (EndMT), an important source of CAFs (Matkar et al., 2016). Finally, CAFs also promotes tumor migration and invasion by inducing EMT of cancers cells (Shan et al., 2017). This EMT induction is carried out through Hedgehog signaling. As above described, NRP1 is a major regulator of Hedgehog signaling. Thus, NRP1 expressed on CAF might also stimulate EMT which increases tumor cell migration and invasion worse prognosis.

### Prognostic Role of NRP1 and NRP2 Pathways

Neuropilins correlate with poor prognosis in many cancers. Here are some examples. NRP1 is overexpressed in bladder cancer and correlates with poor prognosis (Cheng et al., 2014). In osteosarcoma, NRP1 is a prognostic factor of shorter progression-free (PFS) and overall survival (OS) (Zhu et al., 2014). NRP2 contributes to laryngeal squamous cell carcinoma progression and could serve as a new therapeutic target for this type of cancer (Yin et al., 2020). In prostate adenocarcinoma, NRP2 is a marker of bad prognosis (Borkowetz et al., 2020). Some activator of the NRP2 pathway including VEGFC were described as markers of good prognosis in non-metastatic kidney cancers but of poor prognosis in metastatic kidney cancers (Ndiaye et al., 2019). Thus, the level of expression of NRP2 and their partners, has to be determined to adapt a specific therapeutic strategy in tumors at different steps of their development.

### Role in the Therapeutic Response

Resistances to targeted therapies are often related to the activation of alternative tyrosine-kinase receptors-mediated signaling pathways. As above described, NRPs interact with several tyrosine kinase receptors and enhance their activity.

#### Resistance to Chemo- and Radiotherapies

Radio- and chemotherapy are widely used to treat cancers.

A high expression of NRP1 in non-small cell lung cancer cells increases radio-resistance through an ABL-1-mediated up-regulation of RAD51 expression (Hu et al., 2018). In pancreatic cancer, NRP1 increases resistance to gemcitabine and 5-fluorouracil by activating the MAPK signaling pathway (Wey et al., 2005).

The NRP2/VEGFC pathway activates autophagy through the inhibition of mTOR complex 1 activity which helps cancer cells to survive following treatment (Stanton et al., 2013). NRP2 overexpression, induced by SEMA3F in adenocarcinoma, decreases integrin αvβ3 and enhances cell sensitivity to chemotherapy (Zheng et al., 2009).

In some cancers, NRP targeted drug decreases resistance to chemo/radiotherapies.

#### Resistance to Targeted Therapies

In pancreatic ductal adenocarcinoma (PDAC), an increase of active integrin β1 activates AKT signaling and resistance to cetuximab, an anti-EGFR monoclonal antibody (Kim et al., 2017). NRP1-dependent JNK signaling leads to the overexpression of EGFR and IGF1R, which induces resistance to BRAF (melanoma targeted therapy), HER2 (breast cancer targeted therapy) and MET (stomach and lung carcinomas therapy) inhibitors (Rizzolio et al., 2018b).

Neuropilin 2 overexpression decreases EGFR expression and resistance to MET-targeted therapies (Rizzolio et al., 2018a).

Thus, NRPs have become interesting biomarkers to determine the patients’ responsiveness to radio- or chemotherapies or to targeted therapies. Indeed, patients with low NRP1 expression present a better OS than patients with high level of NRP1 (Van Cutsem et al., 2012; Napolitano and Tamagnone, 2019).

Again, combination of targeted therapies to NRP1 inhibitors increase the effects of therapies and reduces resistance.

## Conclusion

Angiogenesis is one of the key mechanisms involved in cancer growth and dissemination. Anti VEGF were approved in combination with standard chemotherapies. Despite an improvement of progression free survival in several types of tumors by anti VEGF treatments, increases in OS were reported. The elevated expression in tumor, endothelial, and immune cells, makes NRP1 and 2 new relevant oncology targets to improve the treatment of cancers. This review describes the different roles and the expression level of NRPs in the different cells constituting the tumor microenvironment. NRPs form holoreceptors with many different receptors and, thus, are involved in many biological phenomena: angiogenesis, lymphangiogenesis, cell proliferation, migration, invasion, and tumor growth. Moreover, NRPs are expressed by several immune cells, in which they exert an activating or inhibiting role on the immune response. In many cancers, NRPs over-expression is synonymous of poor prognosis. This review highlights the implication of NRPs in several hallmarks of cancer and the relevance of targeting the NRPs for the treatment of cancers. Several molecules targeting NRPs are in development: (i) anti-NRP1 antibodies such as the MNRP1685A that has to be optimized to improve the therapeutic window and to decrease its toxic effects; (ii) cyclic, rigid or pseudo-peptides developed by optimizing the sequence ATWLPPR, mimicking the VEGF C-terminal domain interacting with NRP1; (iii) non-peptidic inhibitors such as NRPa-308 that exerts anti-cancer effects in triple negative breast cancer (Liu et al., 2018) and which is currently tested in ccRCC.

Despite these different therapeutic pathways, NRPs targeting must be improved to fight cancers that can benefit the most of these treatments. The antagonist role of NRPs as beneficial or detrimental markers depending on tumor stage suggests cautiousness before administration of anti NRPs treatments.

## Author Contributions

AD and GP were equally responsible for all parts of the manuscript. Both authors contributed to the article and approved the submitted version.

## Conflict of Interest

The authors declare that the research was conducted in the absence of any commercial or financial relationships that could be construed as a potential conflict of interest.
